# The SNF1-type serine-threonine protein kinase *SAPK4 *regulates stress-responsive gene expression in rice

**DOI:** 10.1186/1471-2229-8-49

**Published:** 2008-04-28

**Authors:** Calliste J Diédhiou, Olga V Popova, Karl-Josef Dietz, Dortje Golldack

**Affiliations:** 1 Department of Physiology and Biochemistry of Plants, Faculty of Biology, University of Bielefeld, 33615 Bielefeld, Germany; 2 Gregor Mendel Institute of Molecular Plant Biology, A-1030 Vienna, Austria

## Abstract

**Background:**

Plants respond to extracellularly perceived abiotic stresses such as low temperature, drought, and salinity by activation of complex intracellular signaling cascades that regulate acclimatory biochemical and physiological changes. Protein kinases are major signal transduction factors that have a central role in mediating acclimation to environmental changes in eukaryotic organisms. In this study, we characterized the function of the sucrose nonfermenting 1-related protein kinase2 (SnRK2) *SAPK4 *in the salt stress response of rice.

**Results:**

Translational fusion of *SAPK4 *with the green fluorescent protein (GFP) showed subcellular localization in cytoplasm and nucleus. To examine the role of *SAPK4 *in salt tolerance we generated transgenic rice plants with over-expression of rice *SAPK4 *under control of the CaMV-35S promoter. Induced expression of *SAPK4 *resulted in improved germination, growth and development under salt stress both in seedlings and mature plants. In response to salt stress, the *SAPK4*-overexpressing rice accumulated less Na^+ ^and Cl^- ^and showed improved photosynthesis. *SAPK4*-regulated genes with functions in ion homeostasis and oxidative stress response were identified: the vacuolar H^+^-ATPase, the Na^+^/H^+ ^antiporter *NHX1*, the Cl^- ^channel *OsCLC1 *and a catalase.

**Conclusion:**

Our results show that *SAPK4 *regulates ion homeostasis and growth and development under salinity and suggest function of *SAPK4 *as a regulatory factor in plant salt stress acclimation. Identification of signaling elements involved in stress adaptation in plants presents a powerful approach to identify transcriptional activators of adaptive mechanisms to environmental changes that have the potential to improve tolerance in crop plants.

## Background

Plants respond to abiotic stresses such as cold, drought, and salinity by activation of complex intracellular signaling cascades that regulate biochemical and physiological acclimation. In eukaryotes, protein kinases are key elements involved in signal transduction responsive to metabolism, biotic and abiotic stresses inclusive the major environmental factor salinity. Growth of yeast mutants deficient in the sucrose non-fermenting 1 (SNF1) serine-threonine protein kinase that is related to the mammalian AMP-activated protein kinase was severely inhibited by NaCl indicating a main function of the kinase in regulating adaptative mechanisms to salt stress [1,2]. In plants, a salt-induced mitogen-activated protein kinase (MAPK) has been, for example, identified from alfalfa with SIMK that is activated by the MAPK kinase SIMKK, and involvement of MAPKs in osmotic stress signaling has been shown in tobacco and *A. thaliana *[3-5]. Stress-inducible members within the plant family of serine-threonine protein kinases have been identified within the calcium-dependent protein kinases (CDPKs), the CDPK-related kinases (CRKs), the calmodulin-dependent protein kinases (CaMKs), and the SnRKs that are related to SNF1 from yeast. Members of the SnRK1 subgroup function in regulation of metabolism under environmental stress and have a role in plant development [6-8]. Protein kinases of the SnRK2 and SnRK3 type are specific for plants and implication in ABA signaling was shown for several members of these groups [6,9-11]. *A. thaliana *SnRK3 kinases function in sugar and ABA signaling and in salt stress responses [12-14]. SnRK3 SOS2 interacts with the Ca^2+ ^sensor SOS3 and the plasma membrane Na^+^/H^+ ^antiporter SOS1 involved in regulation of intracellular Na^+ ^homeostasis is activated via the SOS pathway [15].

Important knowledge on stress-inducible signaling pathways has been mainly derived from studies on the stress-sensitive model plants *A. thaliana *and rice whereas regulatory signaling elements have been rarely identified in naturally stress tolerant species.

The experiments of this study characterize the protein kinase *SAPK4 *that was identified in a screen for genes regulated by salt stress in the facultative halotolerant grass *Festuca rubra *ssp. *litoralis *(red fescue). Expressional analyses in the salt-sensitive rice line IR29 showed down-regulation of the *SAPK4 *transcript amounts. Over-expression of the rice *SAPK4 *in rice conferred increased tolerance to salt stress at the seedling stage and in mature plants. In the transgenic rice, Na^+ ^and Cl^- ^accumulation was reduced indicating involvement of *SAPK4 *in regulation of ion homeostasis. The results presented in this study indicate that *SAPK4 *is a determinant of plant salt stress acclimation. Identification of signaling transduction elements that have a role in stress adaptation in naturally stress tolerant plants presents a powerful tool to identify transcriptional regulators of adaptive mechanisms to environmental changes that have the potential to improve tolerance in crop plants.

## Results

### Differences of salt-dependent expression of *SAPK4 *in rice and in *F. rubra *ssp. *litoralis*

**Figure 1:**
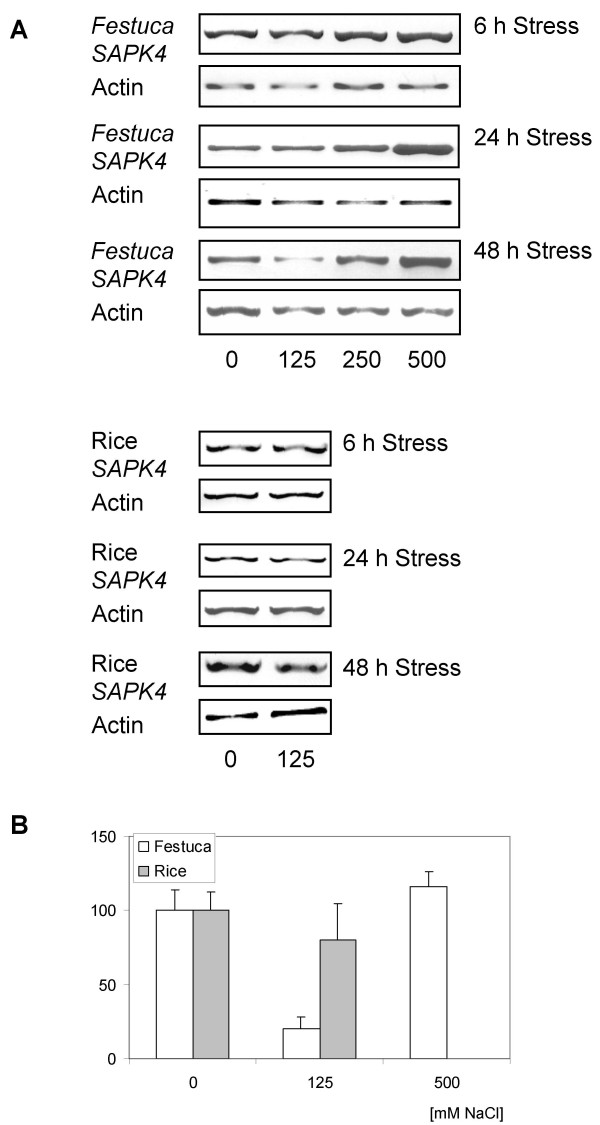
**Effect of salt stress on the transcript abundance of *SAPK4 *in leaves of *F. rubra *ssp. *litoralis *and rice grown under control conditions and treated with NaCl for 6 h, 24 h, and 48 h, respectively.** The transcript levels of *SAPK4 *were quantified by semiquantitative RT-PCR. **(A) **RT-PCR amplification of fragments of the coding region of *SAPK4*. 0 – control, 125 – 125 mM NaCl, 250 – 250 mM NaCl, 500 – 500 mM NaCl. Actin was amplified as a loading control. **(B) **Densitometric analysis of the transcript levels of *SAPK4*. The transcript amounts of *SAPK4 *in leaves of *F. rubra *ssp. *litoralis *and rice grown under control conditions were each set to 100%. The transcript amounts were normalized to actin. Data represent means ± SD. (n = 3).

The study started from a comparative analysis of salt stress-induced transcriptional responses in the salt-sensitive rice variety *Oryza sativa *(ssp. indica) line IR29 and in the salt tolerant grass *F. rubra *ssp. *litoralis*. Genes were identified that differentially respond to salinity in both species. *F. rubra *ssp. *litoralis *is characterized by substantial salt resistance, tolerates up to 500 mM NaCl and continues growth and development with 250 mM NaCl in hydroponic culture (not shown). In contrast, the rice line IR29 is severely damaged by exposure to salt concentrations of 150 mM NaCl [16,17]. The serine-threonine protein kinase *SAPK4 *was identified in a subtracted cDNA library from *F. rubra *ssp. *litoralis *enriched for salt-responsive genes. This experimental approach allowed to identify an EST-sequence from *F. rubra *ssp. *litoralis *that shared 89 and 93% identity on the nucleic acid and amino acid level with *SAPK4 *from rice (not shown). The present study aimed at a detailed analysis of the role of the kinase in plant salt acclimation. By semiquantitative RT-PCR (Fig. 1) and Northern-type RNA hybridizations (not shown), expression of *SAPK4 *was detected in non-stressed control plants of *F. rubra *and rice. In *F. rubra*, salt stress of 125 mM reduced and of 250 mM and 500 mM NaCl increased the transcript level of *SAPK4*. In the salt-sensitive rice line IR29, treatment with 125 mM NaCl for up to 48 h caused a decrease of *SAPK4 *transcript abundance. Due to lethality, 250 mM and 500 mM NaCl were not applied to rice.

**Figure 2:**
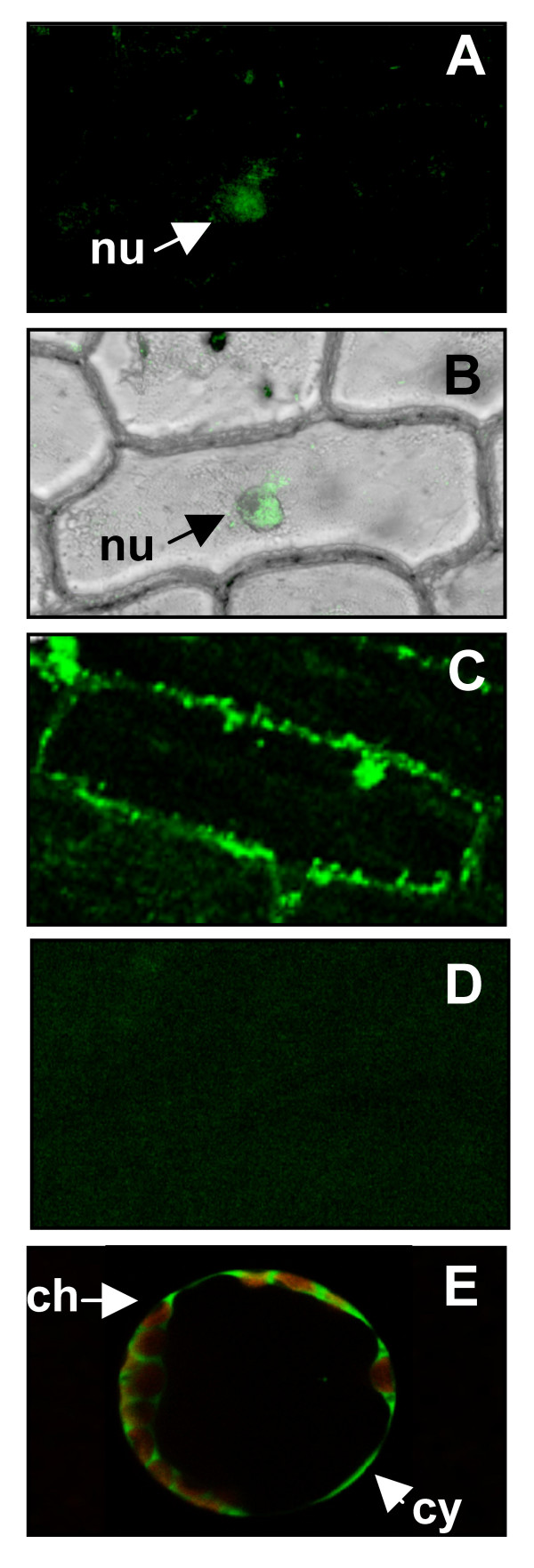
**Subcellular localization of *SAPK4*-protein.(A) **Nuclear localization of *SAPK4*-GFP fusion protein in onion epidermal cells. The arrow points to the nucleus. **(B) **The GFP-derived fluorescence signal of *SAPK4*-GFP fusion protein was merged with a light microscopic image of the transformed onion epidermal cell. **(C) **Onion epidermal cells transformed with a translational construct of GFP as a positive control showed localization throughout the cell with strongest signals in cytoplasm and nucleus. **(D) **Onion epidermal cells transformed with the empty vector as a background control. **(E) **Cytoplasmic localization of SAPK4-GFP fusion proteins in protoplasts of *A. thaliana*. nu – nucleus, ch – chloroplast, cy – cytoplasm.

For an analysis of the subcellular localization of the SAPK4 protein, constructs for the expression of the *SAPK4 *open reading frame cDNA fused to the green fluorescent protein (GFP) reporter gene driven by the 35S-CaMV promoter were generated. Onion epidermis cells were transformed with the translational fusion and fluorescence emission of GFP was monitored under a confocal laser scanning microscope (Fig. 2). In cells incubated for 24 hours in 0.5 × MS nutrient medium, strong GFP signals were detected in the nucleus (Fig. 2A, B). Cells bombarded with the empty vector as a negative control showed no fluorescence (Fig. 2D) As a positive control, onion epidermal cells were transformed with a translational construct of GFP. In this experiment, GFP showed localization throughout the cell with strongest signals in cytoplasm and nucleus (Fig. 2C). To extent the results obtained from the onion epidermal cells system, *A. thaliana *mesophyll protoplasts were isolated and were similarly transformed with the SAPK4-GFP transcriptional constructs. After incubation of transformed protoplasts for 24 hours, GFP-derived fluorescence emission was also detected in the cytoplasmic compartment (Fig. 2E).

### Over-expression of *SAPK4 *in transgenic rice plants

**Figure 3:**
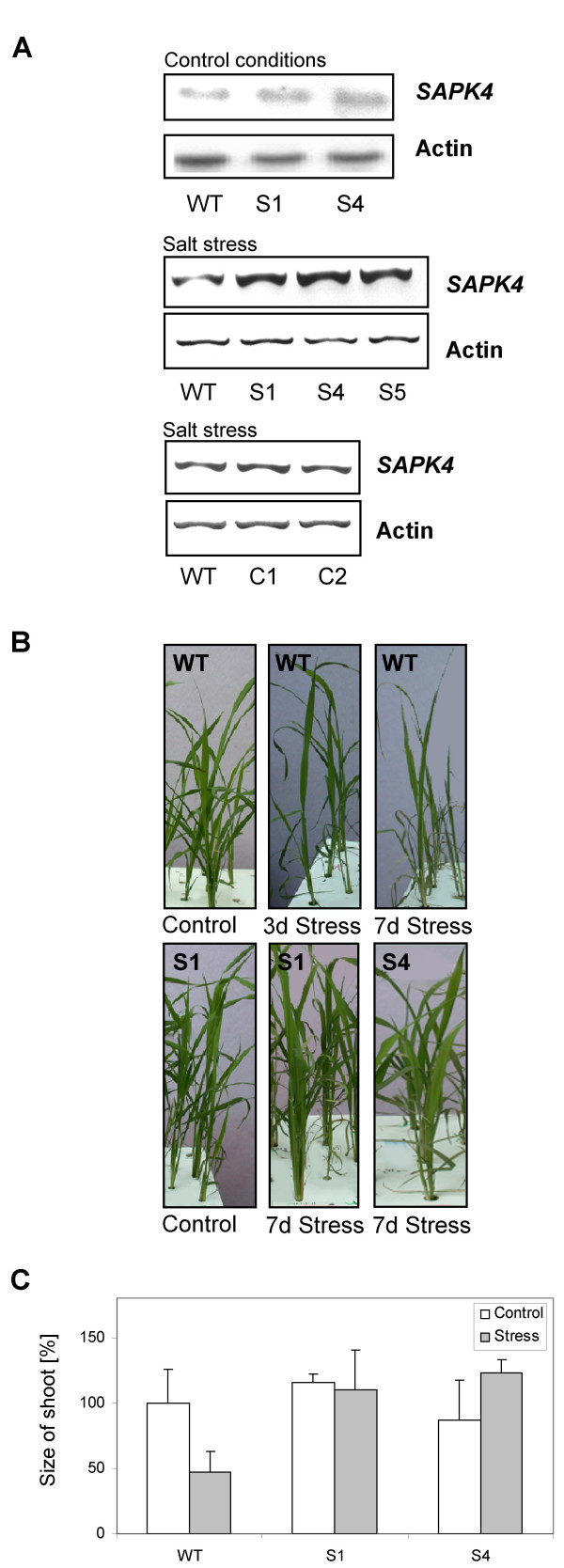
**Increased salt tolerance in transgenic rice plants over-expressing *SAPK4*.(A) **Northern-type hybridization of the expression of *SAPK4 *in leaves of wild-type rice (WT) and the *SAPK4 *over-expressing rice lines S1 and S4 grown under control conditions and analysis of the *SAPK4 *transcript levels by RT-PCR in leaves of wild-type rice (WT) and the *SAPK4 *over-expressing rice lines S1, S4, and S5 exposed to 150 mM NaCl for 48 hours. Analysis of the *SAPK4 *transcript levels by RT-PCR in leaves of wild-type rice (WT) and the rice lines C1 and C2 that were transformed with the empty plant expression vector and that were exposed to 150 mM NaCl for 48 hours is shown as a control. Transcript levels of actin are shown as a loading control. **(B) **Phenotype of wild-type rice and transgenic rice plants over-expressing *SAPK4*. The plants were grown to the age of 8 weeks in hydroponic culture. Control plants and plants that were treated with 150 mM NaCl for up to 7 days. **(C) **Growth performance of wild-type rice and the *SAPK4*-over-expressing lines. Values are means ± S.D. (n = 30).

To generate transgenic rice plants, the salt-sensitive variety IR29 was transformed with vectors containing the open reading frame rice *SAPK4 *cDNA for transcriptional over-expression under control of the 35S-CaMV promoter. Three independent transgenic rice lines designated S1, S4, and S5 were identified by kanamycin resistance and by the presence of the kanamycin resistance gene. Plants of the T2 generations were used for further investigations to examine the role of *SAPK4 *in plant salt tolerance. In non-stressed plants, a moderate increase in the transcript level of *SAPK4 *could be detected in the transgenic lines compared to wild-type rice indicating tight regulation of *SAPK4 *mRNA in rice (Fig. 3). No significant difference between wild-type and transgenic lines was seen in the phenotypes, growth rate, and development up to the age of 12 weeks (not shown). Transgenic plants exposed to elevated NaCl concentrations accumulated more *SAPK4 *transcript compared with wild-type rice (Fig. 3A) and the lines were analyzed for their salt stress responses. In two independent rice lines that were transformed with the empty plant expression vector the transcript amounts of *SAPK4 *were not changed in comparison to non-transformed wild-type rice (Fig. 3) and the phenotype of these plants was not changed as well (not shown). These data demonstrate that the observed effects of *SAPK4 *over-expression in transgenic rice are a result of *SAPK4 *and not of the empty plant expression vector.

### *SAPK4 *is involved in tolerance to salt stress in rice

**Figure 4:**
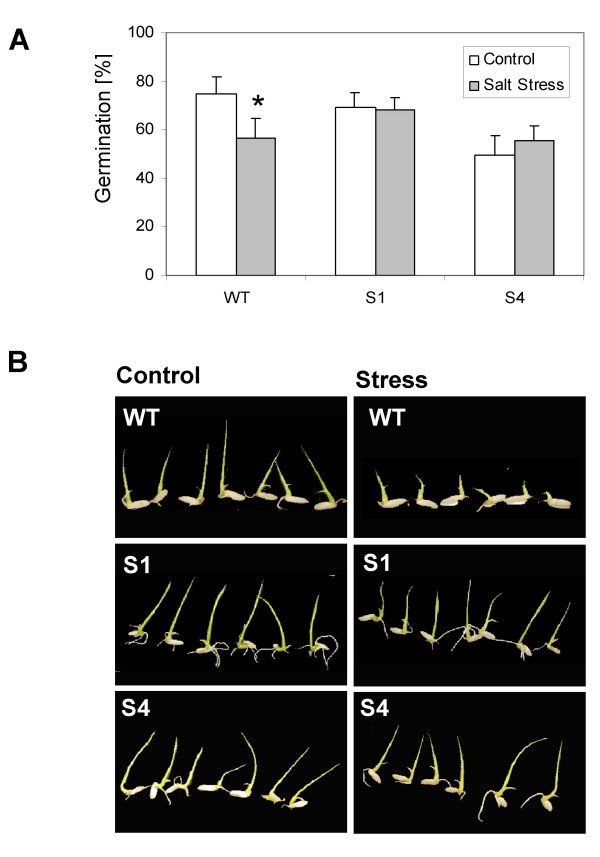
**Increased germination efficiency and seedling development in *SAPK4*-over-expressing rice.(A) **Germination rate of wild-type rice and the *SAPK4 *over-expressing rice lines S1 and S4 under control conditions and after treatment with 50 mM NaCl for 7 days. Values are means ± S.D. (n = 30). * The germination rates of wild type rice under control and under salt stress conditions are significantly different (p < 0.05). **(B) **Phenotype of seedlings grown under control conditions and after treatment with 50 mM NaCl for 7 days.

Wild-type rice and the transgenic lines were grown in hydroponic culture to the age of 3 weeks under control conditions and subsequently were exposed to 150 mM NaCl. At 7 days of salt treatment the transgenic lines displayed improved salt tolerance (Fig. 3B, C). Salt-treated wild-type rice showed growth inhibition and developed chlorosis and necrosis (Fig. 3). In contrast, growth of the transgenic lines was rather unaffected and chlorosis was not apparent (Fig. 3B). To test developmental dependence of enhanced tolerance to increased NaCl concentrations by *SAPK4*, germination assays were performed with wild-type rice and T2 S1 and S4 seedlings. Control and transgenic plants were germinated on control nutrition medium and on medium supplemented with NaCl. Results showed that the germination decreased by approximately 20% in wild-type rice by salt treatment whereas germination was not significantly affected in the transgenic lines (Fig. 4A). In addition, leaf growth was inhibited by the salt stress in wild-type control plants whereas in the transgenic lines the leaf size of seedlings was not significantly changed compared with non-stressed control seedlings (Fig. 4B).

### *SAPK4 *regulates ion accumulation in salt-stressed rice

**Figure 5:**
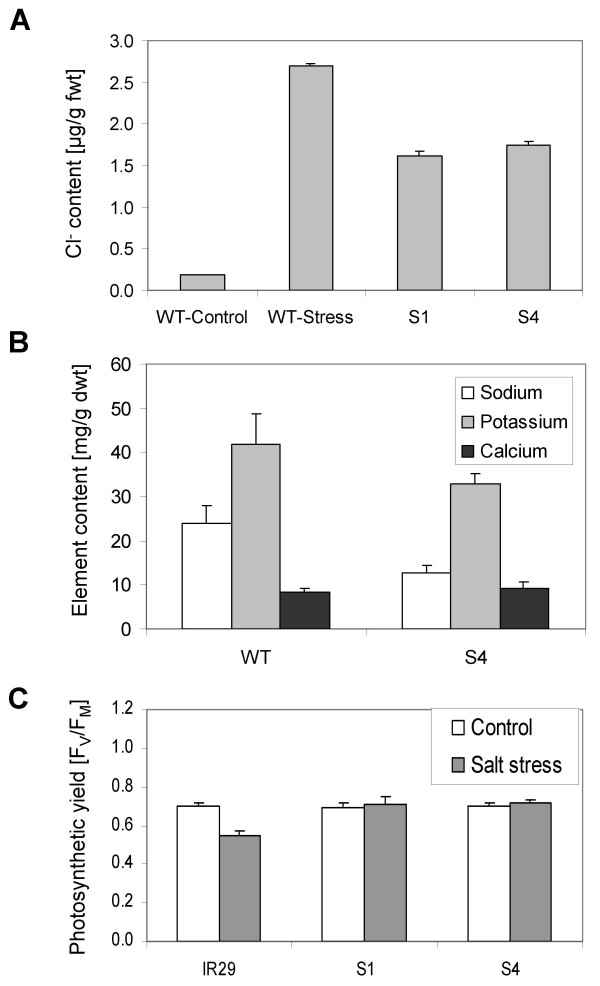
**Ion accumulation and photosynthetic quantum yield ΦPSII in *SAPK4*-over-expressing rice in response to salt stress.(A) **Reduced Cl^- ^content in leaves of 8-week-old *SAPK4*-over-expressing lines S1 and S4 treated with 150 mM NaCl for 48 h compared with wild-type rice grown under control conditions and salt stress. Values are means ± S.D. (n = 30). **(B) **Na^+^, K^+ ^and Ca^2+ ^content in leaves of wild-type rice and the *SAPK4*-over-expressing line S4. The plants were grown in hydroponic culture to the age of 3 weeks and were treated with 150 mM NaCl for 48 h. Values are means ± S.D. (n = 7). **(C) **ΦPSII was calculated from chlorophyll *a *fluorescence. The measurements were performed in attached leaves of 8-week-old control plants and plants treated with 150 mM NaCl for 48 h. Data represent means ± S.D. n = 30.

Accumulation of Cl^- ^was examined in wild-type rice and in the transgenic lines to test whether *SAPK4 *affects accumulation of Cl^- ^under salt stress. Rice plants were grown to the age of 3 weeks and treated with 150 mM NaCl for 48 hours. Under these stress conditions, the lines S1 and S4 contained only 60% of the Cl^- ^contents of wild-type rice (Fig. 5A). Cation homeostasis was addressed by comparing the contents of Na^+^, K^+^, and Ca^2+ ^in wild-type rice and plants of the line S4 that were exposed to the same stress regime as described above. The line S4 accumulated 60% of Na^+ ^and 80% K^+ ^in comparison to wild-type plants whereas no differences in Ca^2+ ^accumulation were observed (Fig. 5B). As a physiological reference chlorophyll *a *fluorescence kinetics were measured and photosynthetic yield calculated. Exposure to 150 mM NaCl for 48 hours resulted in a decreased photosynthetic activity in wild-type rice whereas no significant change occurred in the lines S1 and S4 in comparison with non-stressed control plants of the same lines (Fig. 5C).

**Figure 6:**
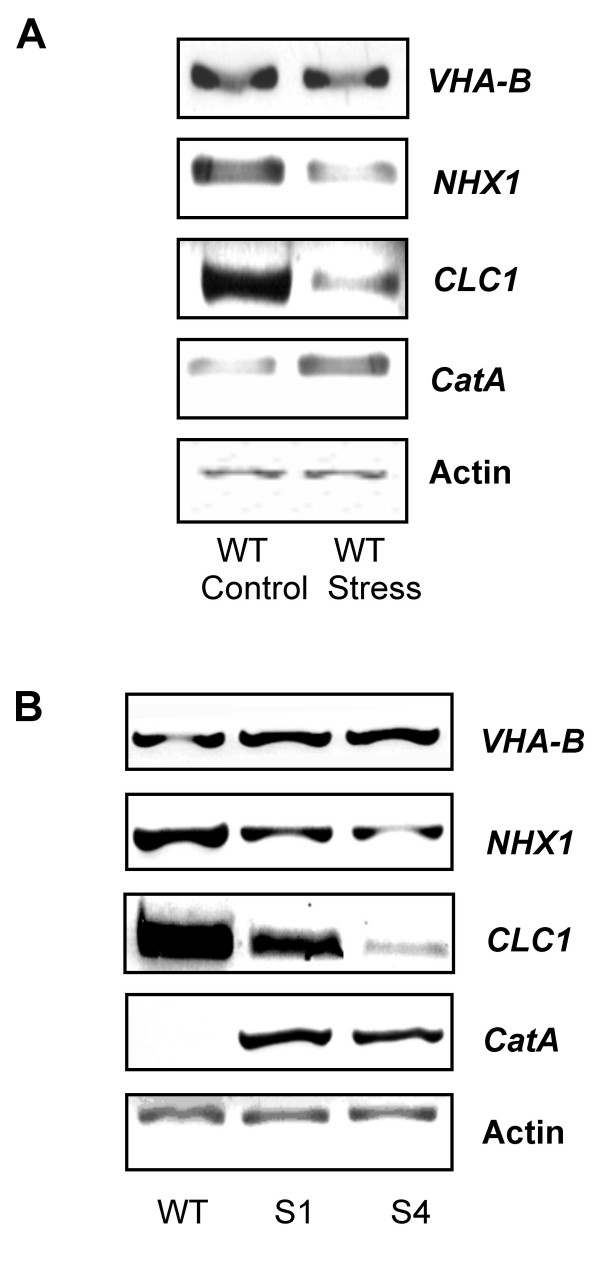
**(A) Transcript accumulation of *SAPK4*-regulated genes in wild-type rice (WT) under control conditions and under salt stress.** Transcript levels were determined by RT-PCR from total RNA isolated from 8-week-old plants. The plants were grown in hydroponic culture and stressed with 150 mM NaCl for 48 h. **(B) **Transcript accumulation of *SAPK4*-regulated genes in wild-type rice (WT) and the *SAPK4*-over-expressing rice lines S1 and S4. Transcript levels were determined by RT-PCR from total RNA isolated from 8-week-old plants. The plants were grown in hydroponic culture and treated with 150 mM NaCl for 48 h. Actin was amplified as a loading control.

The vacuolar ATPase, the vacuolar Na^+^/H^+ ^antiporter *NHX1*, voltage-gated Cl^- ^channels, and catalase are well established targets of salt-dependent regulation (Fig. 6A) and it appeared interesting to study their transcription in wild-type and transgenic rice under salt stress to identify putative target genes regulated by *SAPK4*. The expression levels of the vacuolar ATPase, the vacuolar Na^+^/H^+ ^antiporter *OsNHX1*, the Cl^- ^channel *OsCLC1*, and catalase isozyme A were assessed in wild-type and transgenic rice. The transcript amounts of the V-ATPase subunit B and of the catalase increased by treatment with 100 mM NaCl for 48 hours, whereas the transcript amounts of *OsNHX1 *and *OsCLC1 *decreased in response to NaCl stress (Fig. 6B). In addition, we were interested in analyzing transcription of the plasma membrane Na^+^/H^+ ^antiporter *SOS1 *but were not able to detect its expression in both wild-type and transgenic rice.

## Discussion

Environmental stresses as drought, cold, and salinity limit the agricultural yield of rice that is one of the most important crops. Experimental approaches such as forward and reverse genetics and transcriptome analyses have been chosen to identify molecular key factors that regulate acclimation of rice to environmental changes. Over-expression of the rice transcription factor *OsDREB1A *in *A. thaliana *induced expression of stress-inducible genes and higher tolerance to drought, high-salt, and freezing stresses [18]. Increased salt tolerance of rice was achieved, for example, by transgenic expression of trehalose biosynthetic genes, a Na^+^/H^+ ^antiporter, an aquaporin, the Ca^2+^-dependent protein kinase *OsCDPK7*, and the mitogen-activated protein kinase *OsMAPK5 *[19-23]. A comparison of high-yielding but stress-sensitive rice cultivars with rice varieties with increased stress tolerance indicates that salt-sensitive rice varieties are hampered by delayed stress-induced transcriptional response [24].

In the present study a subtractive cDNA library of the halotolerant grass *F. rubra *ssp. *litoralis *was screened for transcripts that might be regulated differently by salt stress in *F. rubra *ssp. *litoralis *and the salt-sensitive rice line IR29. The identified protein kinase *SAPK4 *belongs to the rice SnRK2 (sucrose nonfermenting 1-related protein kinase2) family and demonstrated that the kinase mediates salt stress signaling in rice. Constitutive over-expression of rice *SAPK4 *conferred increased salt tolerance to rice by interfering with ion homeostasis, maintaining unperturbed photosynthesis and inducing an oxidative stress response. Our results demonstrate that the salt-sensitive crop species rice and the related halotolerant grass *F. rubra *ssp. *litoralis *differ from each other in the salt-dependent regulation of *SAPK4 *that apparently plays a role in salt stress signaling. Improved activation of molecular mechanisms of salt adaptation may be seen in transgenic rice plants over-expressing *SAPK4*. Thus, our results contribute to the understanding of signaling factors that regulate plant salt acclimation. In addition, characterization of *SAPK4 *and identification of target genes that are regulated either directly or by secondary effects by the kinase extends our knowledge on the function of the rice SnRK2 kinase.

The plant SNF1-related kinases (SnRK) that share homology with the yeast SNF1-type kinases have been divided in the three subgroups SnRK1, SnRK2, and SnRK3 based on domain structure [6,7,25]. SnRK1-type proteins have been reported to function in plant development and carbon metabolism such as pollen development in wheat and regulation of enzymes such as an alpha-amylase in wheat embryos and ADP-glucose pyrophosphorylase in potato tubers [26-28]. The wheat SnRK2-subgroup protein PKABA1 is up-regulated by dehydration, cold, and osmotic stress, and involvement in abscisic acid and gibberellin signaling has been shown for the barley homologue [29-32]. Kobayashi et al. [32] analyzed the transcription of *SAPK4 *in leaf blades, sheaths, and roots of 30-days-old rice under control conditions, ABA, NaCl, and mannitol treatment and found increased transcription in roots and blades by treatment with ABA and NaCl. In this work the authors found regulation of SnRK2 family members by phosphorylation [32]. In other studies, in the rice genome 10 SnRK2s could be identified that were activated by hyperosmotic stress, and 3 of the proteins responded to abscisic acid whereas in *A. thaliana *9 of 10 SnRK2s were regulated by hyperosmolarity but not cold indicating function of the kinases in osmotic stress signaling [33,34]. Over-expression of SnRK2.8 improves drought tolerance in *A. thaliana *but did not regulate stomatal movement whereas SnRK2.6 affects ABA-induced stomatal closure [35]. Members of the *A. thaliana *SnRK3 group interact with calcium-binding proteins and have a role in sugar and abscisic acid signaling and in salt stress responses [14].

For a more detailed characterization of rice *SAPK4 *the subcellular partitioning of *SAPK4 *proteins was addressed by localization of GFP fusions and it was found that the protein kinase was distributed in nucleus and cytoplasm. In yeast it has been shown that the beta subunits of the SNF1 kinase regulate its subcellular localization to the nucleus, vacuole, and cytoplasm [36]. Using GFP protein fusions it was shown that SNF1 kinase beta subunits direct the kinase to the nucleus in a glucose-regulated manner [36]. Direct regulatory interaction between signal transduction pathways mediated by the yeast SNF1 kinase and RNA polymerase II holoenzyme has been suggested to activate transcription of glucose-responsive genes [37]. Accordingly, subcellular localization of rice *SAPK4 *in both cytoplasm and nucleus may indicate similar regulatory mechanisms of transcriptional control by the kinase in plant cells.

We generated transgenic rice lines over-expressing *SAPK4*. We found an increased transcript level of *SAPK4 *in comparison to wild-type rice under non-stress control conditions that was, however, more pronounced under salt treatment. A similar effect has been described for other transcripts as well. Shi et al. [46] reported no increased transcript level of the plasma membrane Na^+^/H^+ ^antiporter *SOS1 *in *SOS1*-overexpressing *A. thaliana *when overexpression was driven by the CaMV-35S promoter. The transcript level increased under treatment with NaCl suggesting postranscriptional regulation of *SOS1*. The authors suggested that the *SOS1 *transcript might be unstable in the absence of salt stress and that salt stress causes a stabilization of the *SOS1 *transcript.

Over-expression of *SAPK4 *in transgenic rice plants improved germination, growth and development at both the seedling and the mature plant stage in the presence of increased NaCl concentrations whereas wild-type rice showed severe developmental and physiological inhibition under the same conditions. The *SAPK4 *over-expressing plants accumulated less Na^+ ^and Cl^- ^than salt-stressed wild-type rice in response to salt stress. The K^+^/Na^+ ^ratio was increased in the *SAPK4*-sense plants. In parallel photosynthesis was not impaired in the salt-stressed transgenic rice. Identification of target genes indicates that *SAPK4 *regulates the expression of genes that are known to contribute to ion homeostasis and oxidative stress responses: the vacuolar H^+^-ATPase, the Na^+^/H^+^-antiporter *NHX1*, the Cl^-^channel *OsCLC1*, and a catalase.

The vacuolar H^+^-ATPase mediates electrogenic translocation of protons at endo-membrane compartments of plant cells and energizes processes as cell expansion, secondary activated transport, and adaptation to environmental stress such as salt-induced secondary activated Na^+ ^transport via NHX-type Na^+^/H^+ ^antiporters at the tonoplast [38,39]. Stimulated transcription, translation, and enzyme activity, respectively, is known from halophytes as *Mesembryanthemum crystallinum *and *Suaeda salsa *and, for example, from the V-ATPase subunit A but not subunit D from *A. thaliana *[38,40-42]. Over-expression of the vacuolar NHX1-type Na^+^/H^+ ^transporter that mediates vacuolar Na^+ ^sequestration improved salt tolerance in tomato and rice [43,44]. *SAPK4 *over-expressing rice plants, however, revealed reduced transcript amounts of *OsNHX1 *and a decreased Na^+ ^accumulation indicating that the improved tolerance to salt was caused by cellular Na^+ ^exclusion rather than vacuolar sequestration of the ion. For example, suppression of the Na^+^/K^+ ^co-transporter *HKT1 *reduced Na^+ ^accumulation in wheat roots and resulted in increased salt tolerance [45], and reduced accumulation of Na^+ ^was induced in *A. thaliana *by over-expressing the Na^+^/H^+ ^antiporter *SOS1 *that mediates cellular extrusion of Na^+ ^at the plasma membrane [46]. Voltage-dependent Cl^- ^channels of the CLC-family function in regulation of membrane potential and cellular pH homeostasis, and involvement of plant CLC-type chloride channels in regulation of stomatal movement has been suggested [47]. In rice, expression of the CLC-type channel *OsCLC1 *was analyzed showing salt-dependent transcriptional regulation [48]. In the present work transgenic over-expression of *SAPK4 *in rice repressed both accumulation of Cl^- ^and transcription of *OsCLC1 *indicating involvement of the kinase in regulation of anion homeostasis in salt-treated rice. A role of down-regulation of Cl^- ^channels as *OsCLC1 *in the maintenance of turgor and of the intracellular osmotic potential by restricting Cl^- ^fluxes across the plasma membrane has been hypothesized [48].

In addition to hyperosmotic and hyperionic effects of high salinity, salt-stressed plants are also affected by secondary stresses as excessive generation of reactive oxygen species (ROS). ROS formation is caused by water deficits in salt treated plants that lead to reduced CO_2 _fixation and reduced regeneration of NADP^+ ^in the Calvin cycle [49]. Reactive oxygen species are scavenged by antioxidant metabolites as ascorbate, glutathione, and tocopherols and by detoxifying enzymes as superoxide dismutase, ascorbate peroxidase, and catalase [50-52]. For example, over-expression of glutathione S-transferase and glutathione peroxidase increased growth of transgenic tobacco exposed to salt stress, and transgenic tobacco with reduced catalase activity showed increased susceptibility to salt [53,54]. In this study over-expression of *SAPK4 *was shown to affect expression of a catalase in rice.

## Conclusion

In future investigations it will be interesting to determine the detailed function of this enzyme in the salt acclimation in rice to further advance the understanding of adaptive cellular mechanisms in salt-stressed plants.

**Figure 7:**
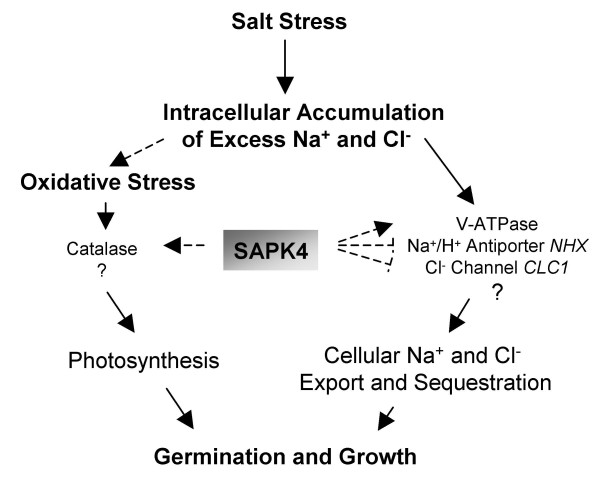
**Model on the putative involvement of the SNF1-type serine-threonine protein kinase *SAPK4 *in the regulation of gene expression in response to salinity.** Catalases are involved in intracellular ROS detoxification and maintenance of photosynthesis [for example 54], the vacuolar ATPase (VHA) energizes the tonoplast NHX-type Na^+^/H^+ ^antiporter for vacuolar Na^+ ^sequestration [55], and transcription of voltage gated Cl^-^-channels is regulated salt-dependently in rice [48].

Summarizing, the results presented in this study demonstrate that *SAPK4 *acts as a regulator of salt acclimation in rice that controls ionic homeostasis and photosynthetic activity and allows continued growth and development in the presence of increased salinity (Fig. 7). The experimental data summarized in the model shown in Fig. 7 are derived from the results of *SAPK4 *over-expression in rice under control of the CaMV 35S promoter performed in the present study. Future experiments using for example *SAPK4 *T-DNA insertion mutants will help to further clarify the functional role of SAPK4 in plant salt adaptation. Identification of salt-inducible signal transduction elements in halotolerant plants and transgenic expression in salt sensitive species as it was performed in this study may be a promising approach to engineer increased resistance to salt stress in crop species.

## Methods

### Plant material, growth conditions, and salt stress

Rice (*Oryza sativa *L. indica) var. IR29 and *Festuca rubra *ssp.*litoralis *were grown in a growth chamber with 14 h light (300 μE m^-2 ^sec^-1^, 25°C) and 10 h dark (21°C) and 50% relative humidity. Seeds were germinated in vermiculite soaked with a modified half-strength Hoagland's nutrition solution [55]. Seedlings were transferred to aerated hydroponic tanks 10 days after germination. For salt stress, the nutrition medium was supplemented with NaCl at a final concentration of 125 and 500 mM. Non-stressed control plants were grown in parallel and harvested at the same time. For transcript analyses, wild-type rice plants were grown to the age of 3 weeks. Experiments with *F. rubra *ssp.*litoralis *were performed at a comparable growth and developmental stage at the age of six weeks. Wild-type and transgenic rice lines were grown to the age of 8 weeks for growth and stress experiments. For germination analyses, seeds of wild-type and of transgenic rice lines were germinated in Petri dishes on sterile filter paper soaked with half-strength Hoagland's nutrition solution. The statistical significance of different germination rates was determined by Student's t-test (p < 0.05). Different NaCl concentrations were chosen for reasons of plant age. Rice plants of the age of 8 weeks were stressed with 125 and 150 mM NaCl that is a severe stress for rice [16]. Germination and growth of seedlings were monitored at 50 mM NaCl. For studies of the transcription of genes regulated by *SAPK4 *the moderate salt stress of 100 mM NaCl was applied.

### Extraction of RNA, Northern hybridization, hybridization of cDNA-arrays, and RT-PCR

Total leaf RNA from rice and of *F. rubra ssp. litoralis *was extracted as described [55]. Northern hybridizations were performed with 20 μg of total RNA per lane [55]. cDNA was synthesized from 5 μg of total RNA with M-MLV RT II [H-] (Promega) and oligo-dT-priming in 20 μl reactions. cDNA probes for Northern detection were generated by PCR with cDNA synthesized from leaves of rice with gene-specific sense and antisense oligonucleotide primers and digoxigenin-dUTP (Roche, Germany) as a label. Transcript analyses were performed for the following genes: *SAPK4 *(AB125305, LOC_Os01g64970; primers for cloning the cDNA: 5'-CACCATGGAGAAGTACGAGGCG-3', 5'-TCATATGCGCAGTGAGCTCAT-3', primers for analyses of transcription: 5'-TGGCTACTCCAAGTCATC-3', 5'-TCGTACTCATCTTCCTCC-3'), *OsNHX1 *(LOC_Os07g47100; primer sequences: 5'-ATCTTCAATGCAGGCTTC-3' and 5'-TGCATCCATCCCAACATA-3'), *OsVHA-B *(LOC_Os06g37180; primer sequences: 5'-ATTGACAGGCAGCTGCAT-3' and 5'-GCAATGTCCATGCTAGGT-3'), *OsCLC1 *(LOC_Os01g65500; primer sequences: 5'-TGTACAAGCAGGACTGGA-3' and 5'-AGATAGGCCTTCACCTCA-3', and catalase isozyme A (LOC_Os02g02400; primer sequences: 5'-GGATGACACCAAGACATG-3', 5'-TCACGTTGAGCCTATTCG-3'). Actin was amplified as a loading control (primer sequences: 5'-GTGATCTCCTTGCTCATACG-3' and 5'-GGNACTGGAATGGTNAAGG-3'). Probes for array hybridization were prepared from each 25 μg of total RNA by incorporating digoxigenin-11-dUTP. Northern blot and cDNA-array membranes were washed with 0.5× SSC at 42°C for 30 minutes and hybridization signals were detected with anti-digoxigenin alkaline phosphatase conjugated Fab fragments and CSPD (Roche, Germany) as a substrate. RT-PCR analyses were performed in standard reactions as described [55]. Actin was hybridized and amplified as a loading control. For densitometric analyses the Gelscan software (INTAS, Germany) was used.

### Construction of subtraction cDNA-library

mRNA was isolated from total RNA with the PolyATract kit (Promega, Mannheim, Germany). A subtraction cDNA-library of F. rubra ssp. litoralis was synthesized with the PCR-Select Kit (Clontech, Heidelberg, Germany) according to the manufacturer's protocol. Same amounts of mRNA from the salt stress treatments of F. rubra ssp. litoralis were pooled for the tester cDNA: 125 mM NaCl, 250 mM NaCl, and 500 mM NaCl for 6 h, 24 h, 48 h, and 7 days at the ages of 5 and 12 weeks, each leaf and root tissue. Same amounts of mRNA from control plants of the same developmental stage and harvested in parallel to the stressed plants were pooled for the driver cDNA. The subtracted cDNA was cloned into pCR-TOPO II (Invitrogen, Karlsruhe, Germany) and the inserts were amplified by PCR with the nested primers 1 and 2R (Clontech). The PCR products were analyzed on agarose gels and products that yielded single bands were selected for further procedures. BLAST analyses of sequenced PCR products (MWG Biotech, SeqLab, Germany) were performed in the rice TIGR database.

### Preparation of cDNA-macroarrays, labeling of probes, and hybridization of cDNA-arrays

The PCR products from the subtraction cDNA-library were purified with QIAquick spin columns (Qiagen, Hilden, Germany) and dissolved in 50% (v/v) DMSO. cDNA-macroarrays with 129 functionally different ESTs were generated at the Center of Genome Research (ZfG) at the University of Bielefeld. The amplified PCR products were transferred in duplicates to nylon membranes (Hybond-N, Amersham Pharmacia Biotech, UK) in a 3 × 3 pattern leaving an empty area in the middle for local background subtraction. The membranes were fixed by baking and UV cross-linking. Labeled detection probes were prepared from each 25 μg RNA with incorporation of digoxigenin-11-dUTP (Roche, Mannheim, Germany) during first strand cDNA synthesis using SuperScript II (Invitrogen) and oligo(dT)-priming. The probes were purified using QIAquick spin columns (Qiagen) to remove unincorporated nucleotides. The probes were denatured at 100°C for 10 min prior to hybridization. The hybridization was performed with the same procedure as described above for Northern hybridizations.

### Generation of constructs and transformation of rice

The open reading frame cDNA of *SAPK4 *was amplified by RT-PCR from rice leaves (var. IR29) with sense and antisense oligonucleotide primers and cloned in pENTR/D-TOPO (Invitrogen, Netherlands). *SAPK4 *cDNA was subcloned in pK2GW7 for over-expression under the control of the constitutive 35S-CaMV promoter and in pK7FWG2 for over-expression with a C-terminal GFP-fusion under the control of the constitutive 35S-CaMV promoter [56]. *Agrobacterium tumefaciens *strain LBA4404 was transformed with the constructs and used for transformation of rice. Mature dehusked rice seeds were sterilized in ethanol (94%, v/v) for 30 seconds, in formaldehyde (0.8%, v/v) for 40 min, and in sodium hypochlorite (1.8%, v/v). The seeds were incubated in Petri dishes containing two layers of sterile Whatman paper and 7 ml sterile deionized water at 42°C for 24 hours to allow pre-germination. The seeds were immersed for 10–15 min in liquid co-culture medium (CCL, modified Chu N6 medium with 2 mg/l naphthalene acetic acid, 1 mg/l kinetin, 55 mM glucose, 100 μM acetosyringone, pH 5,2) containing *A. tumefaciens*. Subsequently, the seeds were transferred into Petri dishes containing solid co-culture medium (CCL with 0.7% (w/v) agarose) and incubated for three days at 25°C in the dark. The seeds were transferred to CCS plates containing cefotaxime (400 mg/l) and were incubated at 25°C under 12/12 h (day/night) photoperiod for three days. To promote tiller and root development, the rice plants were incubated at a photoperiod of 12/12 h (day/night) in N6 medium without hormones plus kanamycin (50 mg/l). Obtained seeds were sterilized as described above and germinated under kanamycin (50 mg/l) treatment. Experiments were performed with transgenic T2 rice plants. Seeds from transformed rice were screened on 0.5 × MS agar medium containing 50 mg/l kanamycin and by RT-PCR of the kanamycin selection marker. Transgenic rice lines transformed with the empty expression vector served as a control.

### Isolation of protoplasts and subcellular localization of SAPK4-GFP fusion proteins

Protoplasts were isolated from *A. thaliana *and were transformed with constructs for expression as described [57]. Transformation of onion epidermal cells was performed according to [57]. For subcellular localization of SAPK4-GFP fusion proteins a confocal laser scanning microscope (Leica DMRE) was used [57].

### Measurement of chlorophyll a fluorescence and of Cl- content

The photosynthetic quantum yield of photosystem II (ΦPSII) was calculated from chlorophyll *a *fluorescence data obtained from attached rice leaves using the Mini PAM (Walz, Germany). The actinic photon flux density was 300 μE m^-2 ^sec^-1 ^as during growth. Steady state maximum fluorescence emission was excited by application of a saturating light pulse (5000 μmol m^-2 ^s^-1^). ΦPSII was calculated according to the manufacturer's instructions. The Cl^- ^content was measured in leaf extracts with a micro-chloro-counter (Marius-Utrecht, Netherlands).

## Authors' contributions

CJD: RT-PCR, CLSM analyses, generation and characterization of transgenic rice lines; OVP: construct generation, CLSM analyses; K–JD: project design and supervision; DG: construct generation, RT-PCR, project design and supervision.
